# Significant Increase in Antibody Titers after the 3rd Booster Dose of the Pfizer–BioNTech mRNA COVID-19 Vaccine in Healthcare Workers in Greece

**DOI:** 10.3390/vaccines10060876

**Published:** 2022-05-30

**Authors:** Konstantina Kontopoulou, Christos T. Nakas, Georgios Papazisis

**Affiliations:** 1 Laboratory of Microbiology, “G. Gennimatas” General Hospital, 54635 Thessaloniki, Greece; ntinakont@yahoo.gr; 2 Laboratory of Biometry, School of Agriculture, University of Thessaly, 38446 Volos, Greece; cnakas@uth.gr; 3 University Institute of Clinical Chemistry, Inselspital, Bern University Hospital, University of Bern, 3010 Bern, Switzerland; 4 Department of Clinical Pharmacology, School of Medicine, Aristotle University of Thessaloniki, 54636 Thessaloniki, Greece; 5 Clinical Research Unit, Special Unit for Biomedical Research and Education, School of Medicine, Aristotle University of Thessaloniki, 56429 Thessaloniki, Greece

**Keywords:** BNT162b2 mRNA COVID-19 vaccine (Comirnaty), immunogenicity, antibody titers, SARS-CoV-2, third dose, booster dose, healthcare workers, Greece

## Abstract

The aim of our study was to assess the immunogenicity of the third dose of the BNT162b2 mRNA COVID-19 vaccine (Comirnaty) in a cohort of 129 health-care workers in Greece whose anti-S1 RBD IgG titers were monitored over the course of nine months. Titers were measured for each participant just before the third dose (nine months after the second dose) and also one month after the third dose. Of the 129 participants, 19 had been previously infected before starting the vaccination scheme. The SARS-CoV-2 IgG II Quant assay on the Architect System was employed to longitudinally assess the titers of IgG against the receptor-binding domain of the S1 subunit of the spike protein (anti-S1 RBD). Boosters raised Geometric Mean Concentrations (GMCs) by a factor of approximately 47 relative to levels at 9 months and by a factor of approximately 23 relative to levels at 6 months. The immune response one month after the third dose was significantly higher than the response achieved one month after the second dose (*p* = 0.008). In conclusion, our findings verify the potent immunogenicity elicited by the third dose in all age and prior COVID-19 status groups, suggesting that the timely administration of the third (booster) dose maximizes the immunogenic potential of the vaccine.

## 1. Introduction

The Pfizer–BioNTech BNT162b2 (Comirnaty) mRNA COVID-19 vaccine, administered as two doses 21 days apart, was authorized for emergency use in Greece in December 2020, in the period when SARS-CoV-2 variant B.1.1.7 was the dominant strain. Since then, marked and sustained declines in the incidence of SARS-CoV-2 infections were observed in all age groups as the percentage of individuals vaccinated with two BNT162b2 doses began to rise, thereby showing, at a national level, the beneficial public health impact of a nationwide vaccination campaign. Real-world evidence from vaccine-rollout programs has shown that COVID-19 vaccines are highly effective against severe disease, hospitalization, and death, and they reduce asymptomatic infection and transmission among family members [1,2].

Despite the initial promising results of the nationwide vaccination campaign, Greece was one of the countries that experienced a resurgence of COVID-19, dominated by the Delta (B.1.617.2) variant of SARS-CoV-2. Due to the greater infectiousness of the Delta variant and the waning immunity after the latest vaccination, a new wave of COVID-19 cases was observed. This was despite the fact that over 66% of the population had been vaccinated with two doses of the available vaccines as of December 2020 [3,4,5].

Although data from the ongoing trial showed that vaccine antibody responses persisted for up to 6 months following the second dose [6], the need to overcome waning immunity and rising rates of COVID-19-related hospital admissions necessitates the administration of an additional vaccine dose. Based on data published, suggesting a pronounced humoral response to a third dose of the mRNA vaccines [7,8], several countries decided to administer a third booster dose of the mRNA COVID-19 vaccine. The Greek Ministry of Health announced a deployment plan in early autumn in order to administer a third dose of the BNT162b2 mRNA COVID-19 vaccine to all adults. This program began with immunocompromised patients in September 2021, and was later expanded to include people aged over 60 years and healthcare workers. The third dose was only given to people who had received the second dose at least 6 months before.

In this study, we aimed to assess the immunogenicity of a third dose of the Pfizer–BioNTech mRNA COVID-19 vaccine (Comirnaty) in a cohort of health-care workers in Greece. Results on the immunogenicity of this vaccine in different time points after the first and the second dose in this cohort have been already published by our group [9,10,11,12].

## 2. Materials and Methods

Serum samples were obtained from a total of 129 healthcare workers one month after the third dose. All participants had previously received a two-dose schedule of the vaccine with a three-week dosing interval. This number reflects the vaccinees willing to have their Ab titer examined for a sixth time and represents a subgroup of the original cohort participating in our previous studies focusing on immunogenicity two weeks after the first dose; two weeks after the second dose; and again 3 months, 6 months, and 9 months after the second dose [9,10,11]. Of the 129 participants in this study, 19 had been infected and recuperated from COVID-19 infection before the first vaccine dose. The trial is registered on the International Standard Randomized Controlled Trial Number registry (study ID: ISRCTN61884303).

Titers of total RBD-specific IgGs against SARS-CoV-2 were determined using the SARS-CoV-2 IgG II Quant assay on the ARCHITECT System (SARS-CoV-2 IgG II Quant, Abbott Sligo, Ireland) on participant-derived serum samples. The SARS-CoV-2 IgG II Quant assay is a chemiluminescent microparticle immunoassay (CMIA) used for the qualitative and quantitative determination of IgG antibodies to SARS-CoV-2 in human serum and plasma on the Alinity and ARCHITECT Systems. The SARS-CoV-2 IgG II Quant assay is used as an aid in evaluating the immune status of infected individuals and to monitor antibody response in individuals who have received the COVID-19 vaccine by quantitatively measuring IgG antibodies against the spike receptor-binding domain (RBD) of SARS-CoV-2.

This highly sensitive and specific SARS-CoV-2 IgG II Quant assay has demonstrated the ability to detect the spike RBD-based vaccine response in longitudinal samples from individuals both with and without prior COVID-19 infection [13]. The use of two-step formats reduces assay non-specific binding (NSB), eliminates the exposure of the conjugate/tracer to potential interferents in the specimen (e.g., human anti-mouse antibodies, ‘HAMA’) and minimizes the potential of high-dose hook effects (prozone effect) [14]. The geometric mean concentration (GMC) and respective 95% confidence intervals were calculated based on the recorded antibody concentration values.

RT-PCR was performed in 18 participants who became infected with SARS-CoV-2, two to five months after the 3rd dose vaccination, and was found to be positive by the AllplexTM SARS-CoV-2 Master Assay [15]. RdRP, (RNA-dependent RNA polymerase) S, E (envelope), N (nucleoprotein) gene, and S variants (any of the HV69/70 deletion, Y144 deletion, E484K, N501Y and P681H) were detected. Following the manufacturer’s instructions, PCR was performed with a CFX96-Dx Touch Real-Time PCR Detection System (Bio-Rad, Hercules, CA, USA) and the results were analyzed with Seegene Viewer V1.0 software (Seegene, Seoul, Korea). Furthermore, the detection of Omicron variant (B.1.1.529) was achieved using AllplexTM SARS-CoV-2 Variants I Assay (HV69/70del E484K N501Y—spike variants) and AllplexTM SARS-CoV-2 Variants II Assay (W152C L452R K417N K417T—spike mutations). Differential diagnosis of SARS-CoV-2 VOC Omicron (B.1.1.529) was based on the following spike gene mutations: HV69/70del, E484A, N501Y and K417N.

Descriptive statistics were based on the geometric means of concentrations (GMC) of anti-SARS-COV-2 spike IgG (AU/mL) followed by the corresponding 95% confidence intervals by groups of interest (gender, age category, previous COVID-19 history). Independent samples t-tests were used for the assessment of differences in log10 IgG levels between groups classified by gender and previous infection with SARS-CoV-2, while one-way ANOVA with Sidak correction for multiplicity was selected for the respective comparisons between age categories. Stata 16.1 (Stata Corp. LLC, College Station, TX, USA) was used for data analysis.

## 3. Results

Our study included 129 participants whose anti-RBD IgG titers were monitored over the course of nine months. Antibody titers were obtained from each participant just before the third vaccine dose (nine months after the second dose) and one month after the third dose. In 100% of our sample, antibody titers exceeded the seropositivity threshold of 50 AU/mL. The overall IgG GMC at nine months, compared to its 6-month value, was reduced by approximately 50% (fold change = 0.489). Boosters raised GMCs by a factor of approximately 23 relative to levels at 6 months (fold change = 22.8) and by a factor of approximately 47 relative to levels at 9 months (fold change = 46.3) (further details are offered in Table 1).

**Figure 1:**
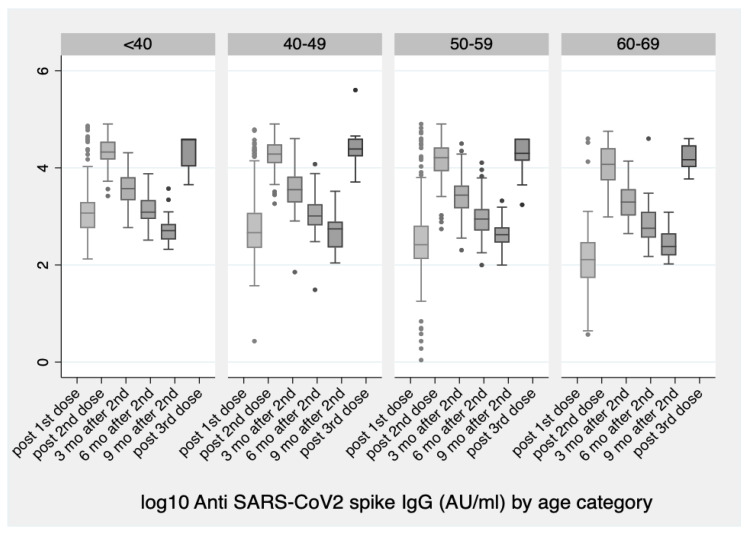
log10 Anti SARS-CoV-2 Spike IgG antibody levels by age group at six selected time points over the course of the vaccination plan: up to three-six-nine months after its completion and one month after the 3rd dose. No significant difference between age groups was found after the third dose (*p* = 0.1).

After the booster dose, there were non-significant differences between females and males (*p* = 0.893) nor between age groups (*p* = 0.294) as opposed to the differences noted in all our previous measurements (Table 1, Figure 1).

**Figure 2:**
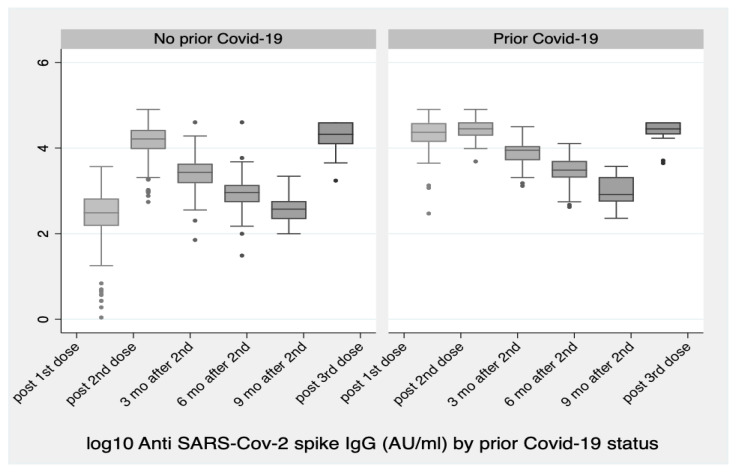
log10 Anti SARS-CoV-2 Spike IgG antibody levels by prior COVID-19 infection status at six selected time points over the course of the vaccination plan: up to three, six and nine months after its completion and one month after the 3rd dose. There was no significant difference between subjects with prior SARS-CoV-2 infection and subjects without history of infection (*p* = 0.155).

Regarding prior COVID-19 infection status, subjects with a prior infection showed higher absolute values in antibody titers after the third dose. Due to the restriction of the upper limit of quantification at 40000 Au/mL and the high number of subjects reaching this (about 27% (30/110) for the no prior COVID-19 group vs. about 42% (8/19) for the prior COVID group), the truncation of measurements at 40000 Au/mL led to a concentration of values near the upper limit for both groups. Formal comparison resulted in a non-significant difference between the two groups (prior/no prior COVID-19 infection) (*p* = 0.155) (Table 1, Figure 2).

**Figure 3:**
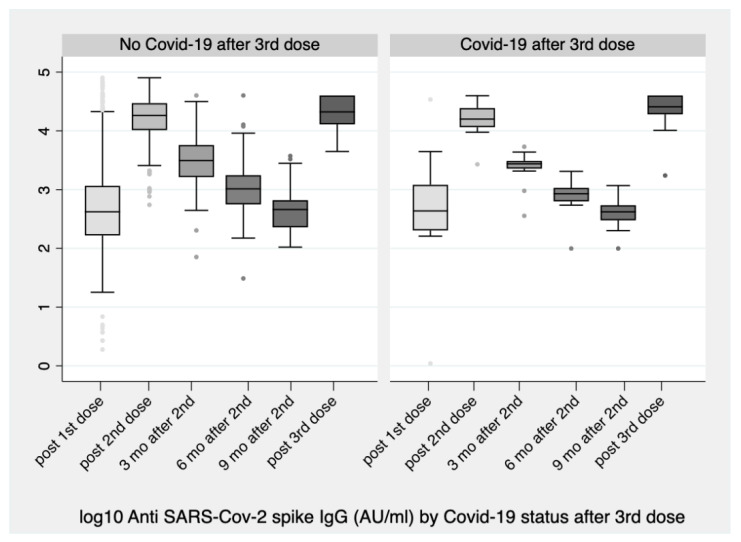
Boxplots of antibody levels (logarithmic scale) of subjects infected 2–3 months after the booster dose versus those not infected. No significant differences were observed.

Eighteen subjects were infected with the Omicron variant (B.1.1.529) two to five months after the third dose. There were consistently non-significant differences between the antibody levels of those infected after the third dose relative to those non-infected (Table 2, Figure 3). Finally, it can be noted that the immune response one month after the third dose was significantly higher than the response achieved one month after the second dose (*p* = 0.008).

Regarding reactogenicity, adverse events were not systematically recorded in our study. The participants were encouraged to spontaneously fill in and submit the Individual Case Safety Report (ICSR) form for any suspect adverse event to the Greek regulatory agency for medicines (EOF), especially regarding the SAEs (serious adverse events) and the SUSARs (suspected unexpected serious adverse events). None of our study participants declared an ICSR submission, thus confirming the absence of serious adverse events in this study.

## 4. Discussion

This is an evaluation of the BNT162b2 vaccine given as a booster to individuals who had been vaccinated 9 months earlier with the same vaccine. Vaccination with a third dose booster elicited higher anti-S1 RBD antibody titers versus titers observed after the primary series vaccinations, suggesting that immune memory was induced by BNT162b2 priming. In particular, our results confirm that one month after the third dose, antibody titers in previously not-infected individuals were significantly higher than one month after the second dose (*p* < 0.001). In previously infected subjects, this difference was not significant (*p* = 0.575). A similar prospective study was conducted in Greece by Terpos et al., where anti-SARS-CoV-2 spike receptor-binding domain IgGs was measured in health professionals. One month after the third dose, the authors found a significant rise in anti-SRBDs of 804% when compared with baseline levels before the booster dose [16,17]

Our results are also comparable with studies suggesting that a third dose of BNT162b2 (Pfizer-BioNTech) COVID-19 vaccine increases antibody levels [18], and that the peak antibody titers post-mRNA booster vaccination were 30/37× higher than pre-booster levels and 11/9× higher than peak levels after the second inoculation [19]. A third dose may be also useful for the prevention of severe disease, hospitalization, and deaths from infections caused by SARS-CoV-2. Indeed, Barda et al. proved that, compared with two doses of the vaccine administered at least 5 months before, receiving a third dose was estimated to have an effectiveness of 93% in preventing COVID-19-related admission to hospital, 92% in preventing severe disease, and 81% in preventing COVID-19-related death [20]. Moreover, in a similar study, Saciuk Y et al. found that the third dose provides added protection against SARS-CoV-2 infection for those vaccinated six months ago [21]. In our study, eighteen participants were infected with the Omicron variant (B.1.1.529), confirmed by molecular techniques, two to five months after the third dose. Due to fifteen mutations in the receptor-binding domain (RBD) that Omicron variant shows, it is suspected that Omicron may have evolved significant immune escape, higher chance of SARS-CoV-2 reinfection, and unprecedented rapid spreading speed [22]. However, more evidence exhibited that three doses of heterologous or homologous booster vaccination led to a 25–100-fold-increase in neutralizing titers compared to two-dose vaccinations [23] thus confirming the necessity of the third dose.

The administration of the third dose is also supported by other studies carried out in Israel. A national study, focusing on the 60-and-over population, found that the rates of confirmed COVID-19 and severe illness were substantially lower among those who received a third dose of the BNT162b2 vaccine [24]. Another study that followed members aged ≥40 years for a maximum 20-day period also found that 7–13 days after the booster shot there was a 48–68% reduction in the odds of testing positive for SARS-CoV-2 infection, and that 14–20 days after the booster the marginal effectiveness increased to 70–84% [25].

The results of a recent VISION Network study from United States, analyzing 222,772 encounters from 383 emergency departments and urgent care clinics and 87,904 hospitalizations from 259 hospitals among adults aged ≥18 years across 10 states, are of paramount importance. The study found that during both the Delta- and Omicron-predominant periods, receipt of a third vaccine dose was highly effective at preventing COVID-19-associated emergency department and urgent care encounters (94 and 82%, respectively), preventing COVID-19—associated hospitalizations (94 and 90%, respectively), and supported strongly the timely administration of the third dose [26].

In terms of side effects, local and systemic adverse events after the booster dose of BNT162b2 were similar to those that occurred after the second dose. The majority of those events were mild or moderate in severity. The most commonly reported side effects included injection site pain and other systematic events such as fatigue, headache, myalgia and arthralgia. It should be noted that limited adverse events of post-vaccination fever were reported.

Our study has certain limitations. We only present data of IgG against RBD of the spike protein, and indicate that the third dose produces a booster in antibody levels; however, neutralizing antibodies and cellular immunity were not measured. Additionally, the measurement of antibody titer six months after the third dose is pending. These limitations may be addressed in further studies.

Overall, our findings verify the potent immunogenicity elicited by the third dose of the BNT162b2 vaccine in all age groups and priorly or not priorly infected subjects, suggesting that the timely administration of the third (booster) dose maximizes the immunogenic potential of the vaccine and possibly protects against new variants of SARS-CoV-2.

## 5. Conclusions

A third dose of the Pfizer–BioNTech mRNA COVID-19 vaccine BNT162b2 (Comirnaty) in health-care workers in Greece administered 9 months after a second dose caused a remarkable increase in the concentration of SARS-CoV-2 anti-S1 RBD antibodies, which declined substantially 9 months after two doses of the BNT162b2 vaccine. The above conclusion indicates that a third dose induces significantly higher antibody titers compared to the first two doses.

## Figures and Tables

**Table 1:** Geometric means of concentrations (GMC)and fold changes of Anti SARS-CoV-2 Spike IgG antibodies six, nine months after two-dose vaccination and one month after the administration of the 3rd dose.

		N	9 Months GMC (95% CI)	9 Months Relative to 6 Months GMC Fold Change (95% CI)	3rd Dose GMC (95% CI)	3rd Dose Relative to 6 Months GMC Fold Change	3rd Dose Relative to 9 Months GMC Fold Change
		129	437.47 (383.34, 499.24)	0.489 (0.447, 0.535)	20,231.52 (18,062.02, 22,661.60)	22.84 (19.77, 26.39)	46.30 (41.09, 52.17)
**SEX**	**Male**	50	464.14 (367.45, 586.26)	0.452 (0.365, 0.558)	20,429.62 (17,014.89, 24,529.66)	20.16 (14.63, 27.79)	44.02 (35.81, 54.10)
	**Female**	79	421.39 (358.81, 494.88)	0.508 (0.464, 0.556)	20,107.13 (17,344.50, 23,309.79)	24.24 (20.80, 28.26)	47.81 (41.23, 55.43)
	***p*-value**		0.429		0.893		
**AGE**	**<40**	19	567.45 (395.85, 813.45)	0.452 (0.386, 0.529)	22,145.50 (15,643.38, 31,350.21)	23.46 (16.47, 33.42)	39.03 (26.28, 57.95)
	**40**–	38	537.71 (407.08, 710.26)	0.458 (0.395, 0.531)	22,948.09 (18,909.49, 27,849.23)	19.59 (14.87, 25.81)	42.84 (33.26, 55.19)
	**50**–	53	413.07 (346.50, 492.44)	0.563 (0.476, 0.665)	19,131.27 (15,858.29, 23,079.75)	24.83 (19.81, 31.12)	46.31 (39.12, 54.83)
	**60**–	19	261.98 (190.83, 359.66)	0.420 (0.328, 0.539)	16791.01 (12,599.75, 22,376.47)	26.20 (16.95, 40.49)	64.09 (51.40, 79.91)
	***p*-value**		0.002		0.294		
**prior COVID status**	**NO**	110	380.49 (335.82, 431.10)	0.503 (0.457, 0.554)	19556.40 (17,304.41, 22,101.46)	26.22 (23.22, 29.62)	51.47 (46.14, 57.41)
	**YES**	19	981.29 (668.44, 1440.58)	0.415 (0.314, 0.548)	24624.25 (17,959.36, 33,762.55)	10.25 (5.75, 18.26)	25.09 (16.09, 39.13)
	***p*-value**		<0.001		0.155		

**Table 2:** GMCs and corresponding 95% CIs of subjects infected after the 3rd dose versus subjects not infected after the 3rd dose over the course of the study. No significant differences were observed.

	COVID after 3rd Dose	N	After 1st Dose	After 2nd Dose	3 mo after 2nd	6 mo after 2nd	9 mo after 2nd	After 3rd Dose
**GMC**	**N**	111	460.57 (318.73, 665.53)	15,585.5 (13,349.04, 18,196.66)	2945.32 (2467.74, 3515.33)	890.06 (740.40, 1069.96)	445.85 (384.56, 516.90)	19871.33 (17,655.59, 22,365.14)
	**Y**	18	464.96 (168.03, 1286.57)	16,433.37 (11,997.75, 22,508.85)	2408.92 (1722.07, 3369.72)	783.01 (549.53, 1115.67)	389.15 (294.80, 513.70)	22,601.53 (15,275.04, 33,442.08)
	***p*-value**		0.985	0.793	0.382	0.573	0.482	0.439

## Data Availability

Not applicable.

## References

[1] Haas E.J., Angulo F.J., McLaughlin J.M., Anis E., Singer S.R., Khan F., Brooks N., Smaja M., Mircus G., Pan K. (2021). Impact and effectiveness of mRNA BNT162b2 vaccine against SARS-CoV-2 infections and COVID-19 cases, hospitalisations, and deaths following a nationwide vaccination campaign in Israel: An observational study using national surveillance data. Lancet.

[2] Pritchard E., Matthews P.C., Stoesser N., Eyre D.W., Gethings O., Vihta K.D., Jones J., House T., VanSteenHouse H., Bell I. (2021). Impact of vaccination on new SARS-CoV-2 infections in the United Kingdom. Nat. Med..

[3] Singanayagam A., Hakki S., Dunning J., Madon K.J., Crone M.A., Koycheva A., Derqui-Fernandez N., Barnett J.L., Whitfield M.G., Varro R. (2022). Community transmission and viral load kinetics of the SARS-CoV-2 delta (B.1.617.2) variant in vaccinated and unvaccinated individuals in the UK: A prospective, longitudinal, cohort study. Lancet Infect. Dis..

[4] Tartof S.Y., Slezak J.M., Fischer H., Hong V., Ackerson B.K., Ranasinghe O.N., Frankland T.B., Ogun O.A., Zamparo J.M., Gray S. (2021). Effectiveness of mRNA BNT162b2 COVID-19 vaccine up to 6 months in a large integrated health system in the USA: A retrospective cohort study. Lancet.

[5] Chemaitelly H., Tang P., Hasan M.R., AlMukdad S., Yassine H.M., Benslimane F.M., Al Khatib H.A., Coyle P., Ayoub H.H., Al Kanaani Z. (2021). Waning of BNT162b2 Vaccine Protection against SARS-CoV-2 Infection in Qatar. N. Engl. J. Med..

[6] Doria-Rose N., Suthar M.S., Makowski M., O’Connell S., McDermott A.B., Flach B., Ledgerwood J.E., Mascola J.R., Graham B.S., Lin B.C. (2021). Antibody Persistence through 6 Months after the Second Dose of mRNA-1273 Vaccine for Covid-19. N. Engl. J. Med..

[7] Ducloux D., Colladant M., Chabannes M., Yannaraki M., Courivaud C. (2021). Humoral response after 3 doses of the BNT162b2 mRNA COVID-19 vaccine in patients on hemodialysis. Kidney Int..

[8] Wu K., Choi A., Koch M., Ma L., Hill A., Nunna N., Huang W., Oestreicher J., Colpitts T., Bennett H. (2021). Preliminary Analysis of Safety and Immunogenicity of a SARS-CoV-2 Variant Vaccine Booster. medRxiv.

[9] Kontopoulou K., Ainatzoglou A., Ifantidou A., Nakas C.T., Gkounti G., Adamopoulos V., Papadopoulos N., Papazisis G. (2021). Immunogenicity after the first dose of the BNT162b2 mRNA Covid-19 vaccine: Real-world evidence from Greek healthcare workers. J. Med. Microbiol..

[10] Kontopoulou K., Ainatzoglou A., Nakas C.T., Ifantidou A., Goudi G., Antoniadou E., Adamopoulos V., Papadopoulos N., Papazisis G. (2021). Second dose of the BNT162b2 mRNA vaccine: Value of timely administration but questionable necessity among the seropositive. Vaccine.

[11] Kontopoulou K., Nakas C., Ntenti C., Katsioulis C., Goulas A., Papazisis G. Antibody Titers 3-Months Post-Vaccination with the Pfizer/Biontech Vaccine in Greece. https://papers.ssrn.com/sol3/papers.cfm?abstract_id=3899094.

[12] Kontopoulou K., Nakas C., Ainatzoglou A., Goudi G., Katsioulis C., Papazisis G. Evolution of Antibody Titers Up to 6 Months Post-Immunization with the BNT162b2 Pfizer/BioNTech Vaccine in Greece. https://papers.ssrn.com/sol3/papers.cfm?abstract_id=3922311.

[13] SARS-CoV-2 Immunoassay (2021). Abbott Core Laboratory (Internet). https://www.corelaboratory.abbott/int/en/offerings/segments/infectious-disease/sars-cov-2-.

[14] Butch A.W. (2000). Dilution protocols for detection of hook effects/prozone phenomenon. Clin. Chem..

[15] Allplex^TM^ 2019-nCoV-2 Master Assay (Cat. No. RV10284X). https://www.fda.gov/media/137178/download.

[16] Terpos E., Karalis V., Sklirou A.D., Apostolakou F., Ntanasis-Stathopoulos I., Bagratuni T., Iconomidou V.A., Malandrakis P., Korompoki E., Papassotiriou I. (2022). Third dose of the BNT162b2 vaccine results in very high levels of neutralizing antibodies against SARS-CoV-2: Results of a prospective study in 150 health professionals in Greece. Am. J. Hematol..

[17] Terpos E., Trougakos I.P., Apostolakou F., Charitaki I., Sklirou A.D., Mavrianou N., Papanagnou E.D., Liacos C.I., Gumeni S., Rentziou G. (2021). Age-dependent and gender-dependent antibody responses against SARS-CoV-2 in health workers and octogenarians after vaccination with the BNT162b2 mRNA vaccine. Am. J. Hematol..

[18] Falsey A.R., Frenck R.W., Walsh E.E., Kitchin N., Absalon J., Gurtman A., Lockhart S., Bailey R., Swanson K.A., Xu X. (2021). SARS-CoV-2 Neutralization with BNT162b2 Vaccine Dose 3. N. Engl. J. Med..

[19] Lau C.S., Phua S.K., Liang Y.L., Oh M.L., Aw T.C. (2022). SARS-CoV-2 Spike and Neutralizing Antibody Kinetics 90 Days after Three Doses of BNT162b2 mRNA COVID-19 Vaccine in Singapore. Vaccines.

[20] Barda N., Dagan N., Cohen C., Hernán M.A., Lipsitch M., Kohane I.S., Reis B.Y., Balicer R.D. (2021). Effectiveness of a third dose of the BNT162b2 mRNA COVID-19 vaccine for preventing severe outcomes in Israel: An observational study. Lancet.

[21] Saciuk Y., Kertes J., Stein N.S., Zohar A.E. (2022). Effectiveness of a Third Dose of BNT162b2 mRNA Vaccine. J. Infect. Dis..

[22] Ai J., Zhang H., Zhang Y., Lin K., Zhang Y., Wu J., Wan Y., Huang Y., Song J., Fu Z. (2022). Omicron variant showed lower neutralizing sensitivity than other SARS-CoV-2 variants to immune sera elicited by vaccines after boost. Emerg. Microbes Infect..

[23] Garcia-Beltran W.F., Denis K.J., Hoelzemer A., Lam E.C., Nitido A.D., Sheehan M.L., Berrios C., Ofoman O., Chang C.C., Hauser B.M. (2022). mRNA-based COVID-19 vaccine boosters induce neutralizing immunity against SARS-CoV-2 Omicron variant. Cell.

[24] Bar-On Y.M., Goldberg Y., Mandel M., Bodenheimer O., Freedman L., Kalkstein N., Mizrahi B., Alroy-Preis S., Ash N., Milo R. (2021). Protection of BNT162b2 Vaccine Booster against Covid-19 in Israel. N. Engl. J. Med..

[25] Patalon T., Gazit S., Pitzer V.E., Prunas O., Warren J.L., Weinberger D.M. (2021). Short term reduction in the odds of testing positive for SARS-CoV-2; A comparison between two doses and three doses of the BNT162b2 vaccine. medRxiv.

[26] Thompson M.G., Natarajan K., Irving S.A., Rowley E.A., Griggs E.P., Gaglani M., Klein N.P., Grannis S.J., DeSilva M.B., Stenehjem E. (2021). Effectiveness of a Third Dose of mRNA Vaccines Against COVID-19–Associated Emergency Department and Urgent Care Encounters and Hospitalizations Among Adults During Periods of Delta and Omicron Variant Predominance—VISION Network, 10 States, August 2021–January 2022. MMWR Morb. Mortal. Wkly. Rep..

